# Study on the Mechanical Properties and Mesoscopic Damage Mechanisms of GGBFS-Modified Recycled Aggregate Concrete Based on Statistical Damage Theory

**DOI:** 10.3390/ma19142990

**Published:** 2026-07-10

**Authors:** Chenyang Yuan, Ziteng Zhang, Weifeng Bai, Jinguang Huang, Junfeng Guan, Yajun Lv

**Affiliations:** 1 School of Water Conservancy, North China University of Water Resources and Electric Power, Zhengzhou 450046, China; yuanchenyang@ncwu.edu.cn (C.Y.); z20231010044@stu.ncwu.edu.cn (Z.Z.); junfengguan@ncwu.edu.cn (J.G.); 2 Henan D.R. Construction Group, Zhengzhou 450000, China; 3 School of Human Settlements, North China University of Water Resources and Electric Power, Zhengzhou 450045, China

**Keywords:** recycled aggregate concrete, ground granulated blast furnace slag, strain rate, mesoscopic damage mechanism, mechanical properties

## Abstract

In order to conduct a comprehensive investigation into the effects of ground granulated blast furnace slag (GGBFS) on the dynamic mechanical properties and mesoscopic damage mechanisms of recycled aggregate concrete (RAC), a combined approach integrating material testing, microscopic characterization techniques, and theoretical analysis was adopted in this study. Two GGBFS replacement rates (0% and 35%) were considered. Uniaxial compression tests were performed to obtain data at different curing ages (*T* = 7 d, 28 d, 56 d, and 150 d) and strain rates ($\dot{\varepsilon }$ = 10^−5^/s, 10^−4^/s, 10^−3^/s, and 10^−2^/s). The obtained data were complemented by nuclear magnetic resonance (NMR) and scanning electron microscopy (SEM) analyses to characterize the evolution of the microstructure and pore characteristics of the specimens. The findings demonstrated that prolonging the curing period continuously densified the microstructure of the specimens, resulting in a commensurate improvement in their initial macro-mechanical behavior. At curing ages exceeding 28 d, the secondary hydration reaction of GGBFS was found to generate additional C-S-H gel, which filled the internal microvoids within the specimens, reduced porosity, and further improved the initial macroscopic mechanical properties. Concurrently, the microstructural characteristics observed at different curing ages, in conjunction with the crack propagation and the fracture toughness effects associated with strain rate, further influenced the initiation, propagation patterns and paths of microcracks during uniaxial compression, as well as the adjustment of the effective stress framework. Furthermore, characteristic parameters describing the evolution of mesoscopic fracturing and yielding damage exhibited regular variations with curing age and strain rate. For specimens cured for 56 d, compared to those with a GGBFS replacement rate of 0%, specimens containing 35% GGBFS exhibited a 4.13% increase in peak stress and a 0.29% decrease in peak strain at $\dot{\varepsilon }$ = 10^−5^/s. At a replacement rate of 35%, as the strain rate increased from $\dot{\varepsilon }$ = 10^−5^/s to $\dot{\varepsilon }$ = 10^−2^/s, the peak stress rose from −50.37 MPa to −60.74 MPa, whereas the peak strain dropped from −23.87 × 10^−4^ to −22.15 × 10^−4^. This study provides significant scientific evidence and a theoretical framework for the engineering application of GGBFS-modified RAC under varying strain rate conditions.

## 1. Introduction

Concrete has historically played a pivotal role in civil engineering applications. Nevertheless, the process of producing concrete releases a considerable amount of CO_2_. This is regarded as a significant contributing factor to the acceleration of global warming. Currently, there is a significant contradiction between the large-scale production and use of concrete and “dual-carbon” goals [1]. At present, a significant quantity of concrete structures is reaching the end of its designated service life, resulting in a substantial increase in the amount of demolished concrete and associated solid waste from construction. These materials are currently disposed of through methods such as open-air dumping or landfilling, which have the potential to contaminate the surrounding environment and exhaust limited land resources [2]. The solution to addressing this challenge is fundamentally rooted in the recycling and repurposing of waste concrete. The use of recycled aggregate concrete (RAC) has proven to be a viable solution, given its capacity to reduce reliance on natural sand and gravel resources, thereby mitigating environmental degradation. Furthermore, it has been demonstrated to be an effective solution to the disposal issue posed by waste concrete [3].

As demonstrated in related studies [4,5,6], recycled concrete aggregate (RCA) exhibits several distinct characteristics that differ from those of natural concrete aggregate (NCA). These include a comparatively lower density, higher water absorption and weaker interfacial transition zones (ITZ). Consequently, the integration of supplementary cementitious materials like ground granulated blast furnace slag (GGBFS), fly ash (FA), metakaolin, etc., into RAC has become a primary research concentration [7]. The utilization of these supplementary materials has been demonstrated to enhance the hydration reaction of cement, thereby effectively mitigating the adverse effects associated with RCA. In their study, Yuan et al. [8] examined the effectiveness of integrating FA with basalt fiber (BF) into pervious concrete made from 100% recycled concrete aggregate (RAPC) to bolster its resistance to freezing. The findings of the study demonstrated that the addition of FA resulted in a significant enhancement of both the mechanical strength and the frost damage resistance of RAPC. Bai et al. [9] enhanced the mechanical properties of RAC by incorporating silica fume into the RAC preparation process, replacing a portion of the cement. Xie et al. [10] examined the effect of using nano-metakaolin in place of traditional silicate cement on the compressive strength of RAC. Their findings revealed that the addition of nano-metakaolin resulted in a reduction in the overall pore volume in RAC and an increase in its compressive strength.

GGBFS, a by-product generated during the process of iron production, is produced in significant quantities worldwide. However, its capacity utilization remains inadequate, underscoring the imperative for research to explore the widespread application of GGBFS in recycled concrete materials. As demonstrated by relevant studies [11,12,13], GGBFS contains elevated levels of active CaO. This has been found to promote the hydration process, leading to the production of C-A-S-H gels. The result of this is a significant enhancement in strength. It can thus be concluded that the incorporation of GGBFS as an additive in concrete mixtures can significantly enhance the mechanical attributes of the concrete. Majhi et al. [14] carried out research on concrete in which GGBFS was employed to substitute various percentages of cement, and RCA was used in place of NCA. The study revealed that an increase in the replacement ratio of RCA or GGBFS weakened the mechanical performance of the concrete. The efficacy of GGBFS in RAC, in comparison to natural aggregate concrete (NAC), demonstrated an enhancement with the progression of the curing age, particularly at 90 d, exhibiting a more substantial increase than at the 7 d and 28 d stages. El-Hassan et al. [15] found that the addition of GGBFS to concrete led to a densification of the concrete matrix and a reduction in its porosity, thereby enhancing the mechanical properties of the concrete. In conclusion, these findings of the research indicated that GGBFS can promote the recycling of construction materials and the low-carbon transformation.

In the context of dynamic loads, such as those induced by earthquakes, the characteristics of concrete can exhibit significant variations when compared with those observed under static loads. The influence of strain rate becomes particularly pronounced under these conditions [16]. Prior studies have demonstrated that the dynamic strength of concrete is significantly influenced by various effects, including the crack propagation and the fracture toughness effects [17,18,19,20,21]. Incorporating strain rate effects is essential for improving the accuracy of experimental data [17]. Zhao et al. [22] observed a more pronounced enhancement in the compressive strength of specimens as strain rate increased. Guo et al. [23] found that both the static and dynamic compressive strengths of RAC showed a decreasing trend with an increasing RCA substitution rate. As demonstrated by Yan et al. [24], an increase in strain rate was observed to be concomitant with intensified damage to the specimens, while concurrently exhibiting an upward trend in dynamic compressive strength. The findings indicated that the strain rate effect influenced the damage mechanism of concrete.

Research has demonstrated that the mechanical performance of concrete is significantly influenced by the curing age of the concrete. In the case of hydraulic concrete, which is subjected to extended periods of curing, its predominant crystalline phase is quartz (SiO_2_). During the hydration process, water reacts with SiO_2_, leading to the creation of a hydrated calcium silicate (C-S-H) gel. As demonstrated in [25], an elevated quantity of C-S-H gels has been shown to enhance the densification of concrete and improve its overall mechanical characteristics. Zhang et al. [26] established that an increase in the curing age was associated with a decrease in the water–cement ratio, and that this, in turn, was linked to an increase in strength growth rate. Bai et al. [27] found that extending the curing age resulted in a more complete hydration process within the specimens, progressively enhancing the peak stress. Concurrently, the peak strain exhibited a downward trend.

As previously mentioned, the mechanical properties of concrete are governed by a multitude of factors, including the composition of the cementitious material, the age of the concrete during curing, and the strain rate. In practical engineering, significant interactions among these factors are typically observed. For instance, the specimen’s microstructural characteristics and compactness at various curing ages are directly governed by the hydration degree of the specimens, which in turn affects the strain rate sensitivity of the material. However, systematic research simultaneously considering these three factors remains relatively limited. Therefore, further exploration and thorough investigation of the evolution of concrete behavior under such multifactorial interactions are required to enable more accurate enhancement of concrete performance [28,29,30].

In addition, although mechanical testing at the macroscopic scale has yielded a substantial amount of stress–strain data, research on the mesoscopic damage mechanisms linking the evolution of microstructure to macroscopic mechanical properties remains limited. Studies at the microscale have focused on the heterogeneous characteristics of internal structure, specifically addressing the microstructure, pore characteristics, microcrack initiation and propagation, and interfacial properties within concrete [31,32]. Such investigations contribute to a deeper understanding of load transmission mechanisms at the microscales and mesoscales, as well as internal damage and fracture processes in concrete. Therefore, the introduction of a mesoscopic statistical damage model is particularly warranted. In such a model, representative volume elements are abstracted into a system of numerous mesoscopic micro-springs or micro-elements whose behavior follows specific probability distributions, including the Weibull or Normal distribution. Through the establishment of statistical evolution equations for micro-element failure, direct descriptions of complex physical damage details are ingeniously avoided, and the cumbersome computational procedures of statistical mechanics are circumvented. Consequently, a physically meaningful quantitative bridge is established between mesoscale damage mechanisms and macroscopic nonlinear stress–strain behavior [9,27].

The present study involved the execution of uniaxial compression experiments on RAC, incorporating GGBFS, under conditions of varying curing periods and strain rates. Nuclear magnetic resonance (NMR) and scanning electron microscopy (SEM) were employed to examine the microstructural development and pore features of the specimens. Furthermore, mesoscopic damage theory was employed to analyze the development of the progression of damage in RAC containing 35% GGBFS during uniaxial compression at different curing ages and strain rates, thereby revealing the intrinsic relationship among microstructural evolution, mesoscopic damage evolution, and macroscopic nonlinear stress–strain behavior. In this paper, tensile stress and tensile strain are considered positive, while compressive stress and compressive strain are considered negative.

## 2. Experimental Investigations

### 2.1. Raw Materials

**Figure 1:**
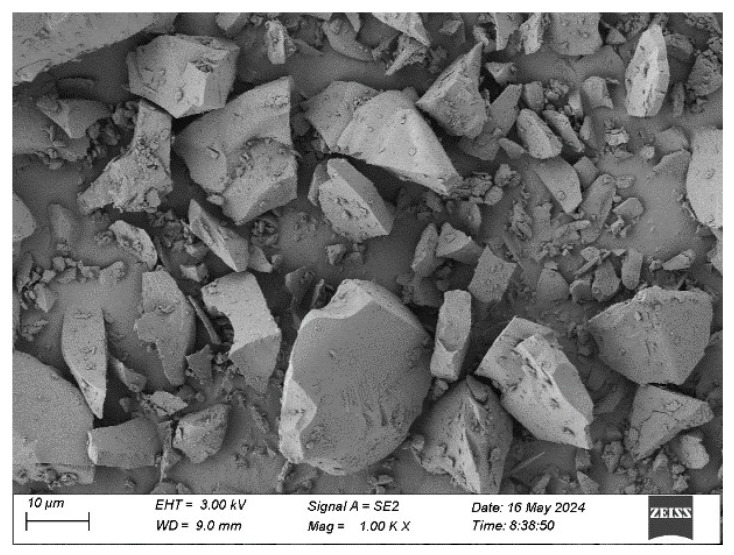
SEM images of GGBFS.

Figure 1 presents SEM images of GGBFS (From Yantai Anbao Environmental Protection Technology Co., Ltd., Yantai, China), revealing distinctive block-like structures of varying sizes and shapes. The main chemical components of ordinary Portland cement (grade P.O 42.5) and GGBFS are listed in Table 1. The GGBFS used in this study meets Grade S95 requirements (specific surface area is 450 m^2^/kg), with a 7-day activity index of 78%, a 28-day activity index of 96%, and a median particle size D50 of approximately 12–18 μm. A comparison of the chemical compositions shows that GGBFS contains higher levels of SiO_2_, Al_2_O_3_, and MgO, but a lower CaO content than cement.

**Figure 2:**
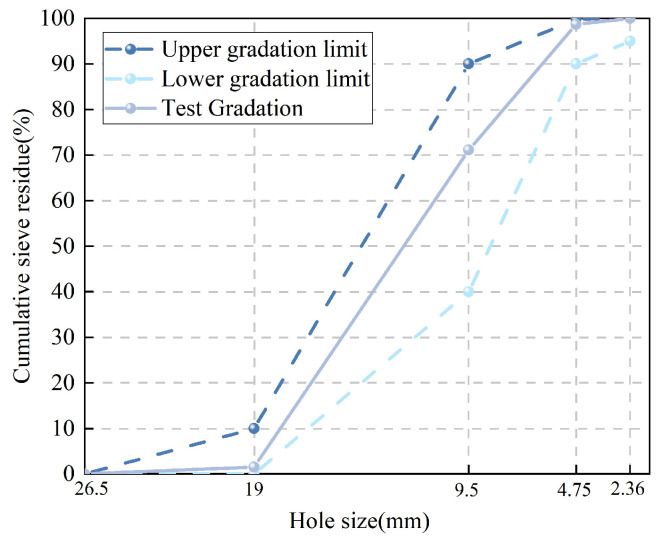
RCA particle grading.

The coarse aggregate was RCA with a particle size range of 5–20 mm. The RCA was obtained from demolished buildings in Shaozhuang, Zhengzhou. The construction waste was coarsely crushed at the demolition site, mixed with impurities such as dust, glass, and thread fragments, and then transported to the laboratory, where it was re-crushed, sieved, and repeatedly washed before use. The fine aggregate (Zhengzhou City, China) was natural river sand with a fineness modulus of 2.8. The particle size distribution and performance indices of the RCA are shown in Figure 2 and Table 2. All procedures conformed to the Chinese standard test method (SL/T 352-2020) [33], and tap water was (Zhengzhou, China) used throughout the experiments.

### 2.2. Mix Proportion Design and Specimen Preparation

The detailed mix ratios of RAC with varying amounts of GGBFS replacement are presented in Table 3. BFS0 was a concrete specimen in which cement served as the cementitious material and RCA as the aggregate. Existing research has determined that the optimal GGBFS replacement rate is 35% [34]. Given that this study aimed to investigate the effects of curing age and strain rate on the mechanical properties and mesoscopic damage mechanisms of GGBFS-modified RAC, two replacement rates—0% and 35%—were selected as the reference control group and the optimally modified group, respectively, to meet the requirements of the experimental design. The detailed summary table of the sample quantities is shown in Table 4. The water absorption of RCA was found to be relatively high, which was attributed to the presence of residue from old cement mortar on its surface [35,36,37]. Consequently, the preparation of RAC necessitated the incorporation of additional water. Using the measured water content and water absorption of RCA, the additional water requirement was calculated to be 8.6 kg/m^3^ [38]. Furthermore, a polycarboxylic acid water-reducing agent was also incorporated into the mix. This agent exhibited optimal water reduction and water retention properties, thereby enhancing the workability of concrete. The mixing amount of this agent was set at 0.25%.

First, the inner surface of the HJW-100 Concrete Mixer was dampened to avoid moisture absorption from the mixture during the mixing process. The procedure for producing concrete involved the following steps. Firstly, the RCA was combined with additional water. Then, the materials (cement, sand, RCA, and GGBFS) were poured into the mixer and mixed for a period of three minutes to ensure adequate dispersion. Following this, the mixer was supplemented with water and a water-reducing agent, and the blending process was continued for a further three minutes to achieve a uniform mixture. Subsequent to the conclusion of the mixing process, a cubic mold with 100 mm side lengths was filled with the mixture. The vibration table was utilized to position the mold onto it, and oscillation was applied to ensure that the specimens were adequately filled. All specimens were removed from their molds after a period of 24 h at ambient temperature and moved to a standard curing chambers (20 ± 2 °C temperature and 95 ± 2% humidity) for curing.

### 2.3. Test Methods

The specimens were subjected to uniaxial compression loading through a pressure testing machine (YAW-5000, Shenzhen Sizhi Zongheng Technology Co., Ltd., Shenzhen, China). The specific experimental procedure was identical to that of the preceding experiment [9,27,34]. The effect of various curing ages (*T* = 7 d, *T* = 14 d, *T* = 28 d, *T* = 56 d, *T* = 90 d, and *T* = 150 d) of the test blocks at the same strain rate (0.18 mm/min) was first accomplished. In particular, the relationship between the loading rate and the strain rate is shown in Equation 1. Subsequently, different strain rate tests were conducted at rates (0.06 mm/min, 0.6 mm/min, 6 mm/min, and 60 mm/min) for various curing ages (*T* = 7 d, *T* = 28 d, *T* = 56 d, and *T* = 150 d). The apparatus was stopped after the specimens failed. Stress–strain curves were subsequently obtained for all specimens. For each group of four specimens that were tested in parallel, the resultant test value was discarded if it deviated from the mean by more than 15%. The compressive strength for each group was then determined as the average of the three retained specimens.

(1) $$\dot{\varepsilon }=\frac{V}{L_{0}}$$

In this equation, $\dot{\varepsilon }$ represents the strain rate (/s), V represents the loading rate (mm/min), and $L_{0}$ represents the specimen length (mm).

The experimental testing was conducted using nuclear magnetic resonance (NMR) equipment (MesoMR12-060H-I, Suzhou Nymai Analytical Instruments Co., Ltd., Suzhou, China). The specimens were retrieved for the NMR test after they had reached the specified curing age. Prior to conducting the test, the NMR machine was calibrated using a standard porosity specimen. The center frequency and pulse width were adjusted, and markers were created for the test. Initially, the test block was immersed in water under vacuum pressure for a period of 24 h, thereby achieving a water-saturated state. Subsequently, the test block was retrieved and the residual surface water was meticulously removed. The specimens were analyzed using nuclear magnetic resonance (NMR) relaxometry to determine their pore size distribution. This required the samples to be positioned within the instrument and a complete scan to be performed.

The present study set out to explore the influence of GGBFS substitution ratios on microstructural development. A selection of fragments was subjected to vacuum drying, gold coating, and subsequent examination using a Zeiss Sigma 300 scanning electron microscope (Carl Zeiss Microscopy GmbH, Oberkochen, Germany).

## 3. Results and Discussion

### 3.1. Uniaxial Compression

#### 3.1.1. Peak Stress

**Figure 3:**
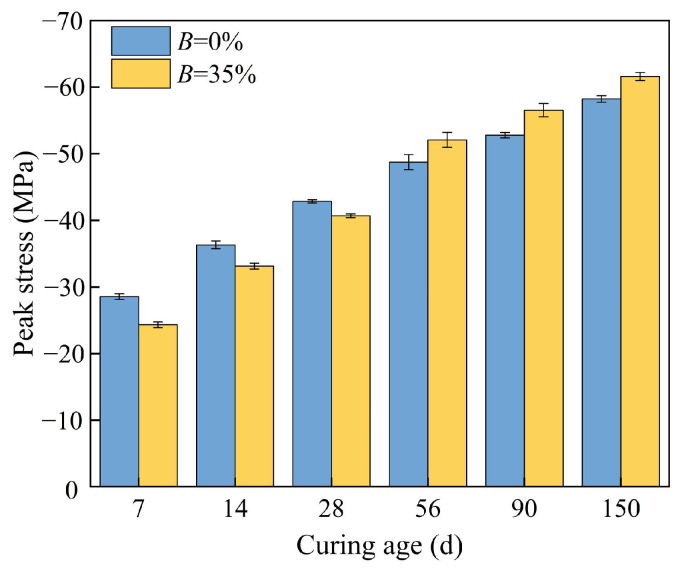
Peak stress.

**Figure 4:**
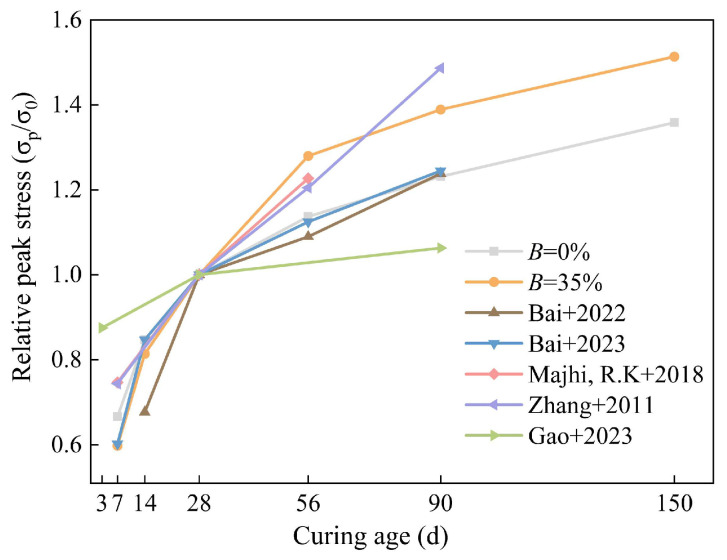
Relative peak stress. Bai et al., 2022 [9]; Bai et al., 2023 [27]; Majhi, R.K et al., 2018 [14]; Zhang et al., 2011 [26]; Gao et al., 2023 [7].

As demonstrated in Figure 3, the relationship between different curing ages and peak stresses of RAC specimens for two GGBFS replacements was illustrated. The experimental findings revealed that the peak stress in the RAC specimens, which had two different replacement levels, demonstrated a rising pattern as the curing age was prolonged. For specimens with the same replacement amount, the maximum peak stress was attained at 150 d. When the curing age reached 7 d, the peak stresses of BFS0 specimens and BFS35 specimens were −28.57 MPa and −24.34 MPa, respectively. After this, as the curing age increased to 14 d, 28 d, 56 d, 90 d, and 150 d, the peak stress of BFS0 specimens exhibited an increase of 27.16%, 50.05%, 70.63%, 85.40%, and 103.85%, respectively; the peak stress of BFS35 specimens exhibited an increase of 36.11%, 67.21%, 112.57%, 132.29%, and 153.12%, respectively. The observed growth rates indicated that the compressive strength of both specimen types was significantly affected at early ages, with the relative increase decelerating after 28 d. Comparing the two RAC specimens at identical curing ages showed that, before 28 days, BFS35 specimens exhibited a lower peak stress relative to BFS0 specimens. However, after a duration of 56 days, the BFS35 specimens exhibited a higher peak stress in comparison to the BFS0 specimens. The underlying cause of this phenomenon was attributed to the primary function of GGBFS, which was to enhance the strength of concrete in the later stages of its curing [39,40]. The blend of the RAC with GGBFS demonstrated a substantial increase in strength beyond 28 days, which was primarily attributable to the activation of GGBFS. Consequently, a substantial quantity of C-S-H gel was formed [41,42,43,44,45]. The relative values of peak stress (*σ*_p_/*σ*_0_) with curing age from several literature sources are presented in Figure 4, where *σ*_0_ is the corresponding peak stress at the age of 28 d. The results showed a consistent pattern of peak stresses rising over time. The findings demonstrated that the strength of concrete is significantly influenced by the curing age during the initial 28 days. Subsequent to this period, the rate of strength gain declined progressively.

**Figure 5:**
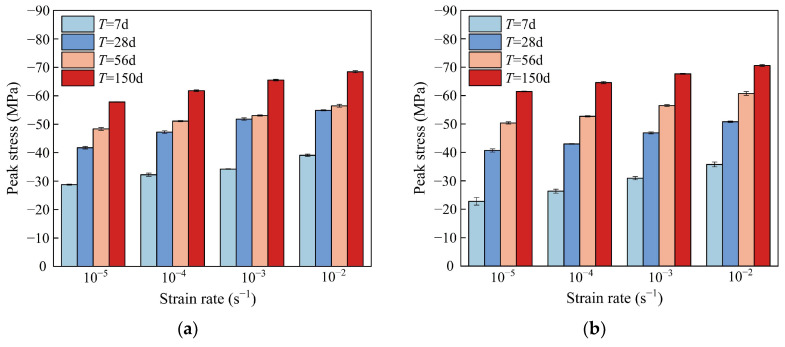
Relationship between peak stress and strain rate. (**a**) *B* = 0%. (**b**) *B* = 35%.

The peak stress diagram for the BFS0 and BFS35 specimens at different curing ages and strain rates is shown in Figure 5. Taking $\dot{\varepsilon }$ = 10^−5^/s as an example, for the BFS0 reference group (without GGBFS), the peak stress was observed to increase from −28.8 MPa to −57.83 MPa as the curing age increased from 7 d to 150 d; correspondingly, the peak stress of the BFS35 specimens increased from −22.78 MPa to −61.43 MPa over the same curing age interval. In the early stage (T ≤ 28 d), a 2.54–20.9% reduction in peak stress was recorded for the BFS35 specimens compared to the BFS0 reference group, which was attributed to the relatively limited degree of hydration in the cementitious system. However, once the curing age exceeded 28 d, a 4.13–6.23% increase in peak stress was observed for the BFS35 specimens relative to the BFS0 specimens. The enhancements in question were ascribed to the sustained secondary hydration reaction of GGBFS and the filling of voids by hydration products. This process optimizes the microstructure of the ITZ, thereby enhancing the macroscopic mechanical properties of the concrete under prolonged curing age conditions.

In the context of the prevailing curing age conditions, a consistent and progressive escalation in peak stress was observed as the strain rate increased, a phenomenon that was evident across all groups. Taking *T* = 56 d as an example, as the strain rate was increased from $\dot{\varepsilon }$ = 10^−5^/s to $\dot{\varepsilon }$ = 10^−2^/s, the peak stress of the BFS0 specimen was found to increase from −48.37 MPa to −56.49 MPa, corresponding to a 16.79% increase; correspondingly, under the same condition, the peak stress of the BFS35 specimen was observed to increase from −50.37 MPa to −60.74 MPa, corresponding to a 20.59% increase. Under the influence of mechanisms such as crack propagation and fracture toughness effects, significant strain rate sensitivity was exhibited by specimens in all groups. The underlying mechanisms are proposed as follows: A significant time constraint on the initiation and propagation of microcracks is imposed by elevated strain-rate loading, thereby forcing the cracks to nucleate and propagate along more energy-dissipative pathways; this manifests macroscopically as an increase in peak stress. It has been demonstrated that higher strain rates (compared to quasi-static) induce significant viscous resistance, originating from free water films within narrow pores or microcracks. Furthermore, this resistance has been shown to be positively correlated with crack opening velocity. The physical separation of the crack surfaces is effectively delayed by the crack propagation and the fracture toughness effects, whereby the apparent resistance of the matrix to failure is enhanced. Moreover, GGBFS incorporation further heightened the strain rate sensitivity of the specimens. At curing ages of 7 d, 28 d, and 150 d, respectively, increases in peak stress of 57.02%, 24.87%, and 14.94% were observed for the BFS35 specimens as the strain rate was increased from $\dot{\varepsilon }$ = 10^−5^/s to $\dot{\varepsilon }$ = 10^−2^/s. For BFS0 and BFS35 specimens at different curing ages and different strain rates, the standard deviation ranges of the peak stress were 0.07–0.57 and 0.11–1.35, respectively.

**Figure 6:**
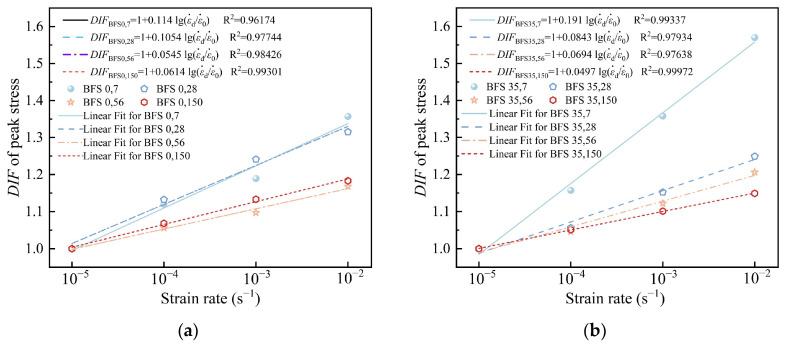
Dynamic increase factor of peak stress. (**a**) *B* = 0%. (**b**) *B* = 35%.

Figure 6 illustrates the evolution of the dynamic strength enhancement factor (DIF) with curing age for both BFS0 and BFS35 specimens, thereby providing a more quantitative assessment of how strain rate influences peak stress [46]. The notation “BFS0,7” is used to denote the BFS0 specimen at a curing age of 7 d; the same convention was applied to the other cases. The analysis indicated that at curing ages of 7 d, 28 d, 56 d, and 150 d, peak stress increases of 11.4%, 10.54%, 5.45%, and 6.14%, respectively, were observed for the BFS0 specimens when the strain rate was increased tenfold relative to the reference value of $\dot{\varepsilon }$ = 10^−5^/s; for the BFS35 specimens, the corresponding increases were 19.1%, 8.43%, 6.94%, and 4.97%, respectively. Thus, strain rate sensitivity was exhibited by both groups, and a linearly increasing trend in DIF was observed as the strain rate transitioned from quasi-static to moderate conditions. It is noteworthy that the increase in DIF was most significant for both groups at a curing age of 7 d, and the strain rate sensitivity was particularly pronounced in the BFS35 specimens in the early stages.

#### 3.1.2. Elastic Modulus

**Figure 7:**
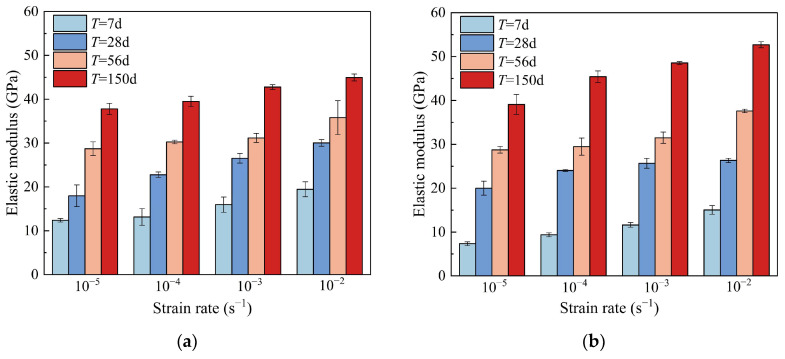
Relationship between elastic modulus and strain rate. (**a**) *B* = 0%. (**b**) *B* = 35%.

The elastic modulus diagram for the BFS0 and BFS35 specimens at different curing ages and strain rates is shown in Figure 7. Taking $\dot{\varepsilon }$ = 10^−5^/s as an example, for the BFS0 reference group (without GGBFS), the elastic modulus was observed to increase from 12.38 GPa to 37.80 GPa as the curing age was increased from 7 d to 150 d; correspondingly, an increase from 7.35 GPa to 39.08 GPa was recorded for the BFS35 specimens. It is noteworthy that at early curing ages, the BFS35 specimens demonstrated a significantly lower elastic modulus compared with the BFS0 reference group.

Under the same curing age conditions, a monotonically increasing trend in elastic modulus as the strain rate increased was observed for all specimens. Taking *T* = 56 d as an example, as the strain rate was increased from $\dot{\varepsilon }$ = 10^−5^/s to $\dot{\varepsilon }$ = 10^−2^/s, the elastic modulus of the BFS0 specimen was observed to increase from 28.83 GPa to 35.80 GPa, corresponding to a 24.18% increase; similarly, an increase from 28.75 GPa to 37.61 GPa was recorded for the BFS35 specimen, corresponding to a 30.82% increase. The findings indicated that the incorporation of GGBFS led to an enhancement in the sensitivity of the elastic modulus to the strain rate. For BFS0 and BFS35 specimens at different curing ages and different strain rates, the standard deviation ranges of the modulus of elasticity were 0.4–4.79 and 0.23–6.49, respectively.

#### 3.1.3. Peak Strain

**Figure 8:**
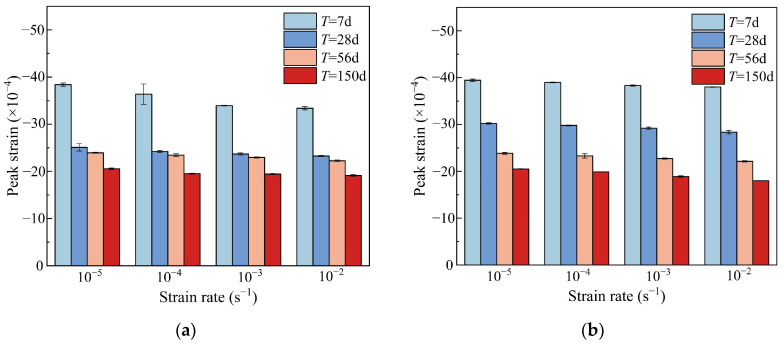
Relation between peak strain and strain rate. (**a**) *B* = 0%. (**b**) *B* = 35%.

The peak strain diagram for the BFS0 and BFS35 specimens at different curing ages and strain rates is shown in Figure 8. Taking $\dot{\varepsilon }$ = 10^−5^/s as an example, for the BFS0 reference group (without GGBFS), the peak strain was observed to decrease from −38.39 × 10^−4^ to −20.57 × 10^−4^ as the curing age was increased from 7 d to 150 d; correspondingly, a decrease from −39.46 × 10^−4^ to −20.52 × 10^−4^ was recorded for the BFS35 specimens. Remarkably, the BFS35 specimens exhibited a higher peak strain relative to the BFS0 group at early curing ages.

Under the same curing age conditions, a monotonically decreasing trend in peak strain with strain rate increased was observed for each group. Taking *T* = 56 d as an example, as the strain rate was increased from $\dot{\varepsilon }$ = 10^−5^/s to $\dot{\varepsilon }$ = 10^−2^/s, the peak strain of the BFS0 specimen was observed to decrease from −23.94 × 10^−4^ to −22.25 × 10^−4^, corresponding to a decrease of 7.06%. A similar trend was noted for the BFS35 specimen, which exhibited a decrease from −23.87 × 10^−4^ to −22.15 × 10^−4^ was recorded for the BFS35 specimen, corresponding to a decrease of 7.21%. These findings revealed that the addition of GGBFS further amplified the sensitivity of peak strain to strain rate. For BFS0 and BFS35 specimens at different curing ages and different strain rates, the standard deviation ranges of the peak strain were 0.07–2.15 and 0.03–0.46, respectively.

#### 3.1.4. Stress–Strain Curves

**Figure 9:**
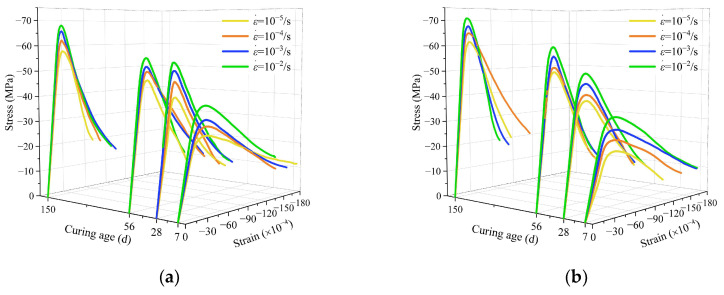
Uniaxial compressive stress–strain curves at different strain rates. (**a**) *B* = 0%. (**b**) *B* = 35%.

As illustrated in Figure 9, the uniaxial compressive stress–strain curves of RAC with GGBFS replacement ratios of 0% and 35% are presented. Across the range of curing ages, the stress–strain curves of both specimens demonstrated a comparable shape. The analysis revealed two distinct phases in the observed curves: an ascending phase and a descending phase. Both phases were characterized by smooth and continuous transitions. In the context of constant strain rate conditions, a notable alteration in curve characteristics was observed with extended curing age. This was characterized by an augmented slope in the ascending phase and a shift in the peak point towards the upper left. This shift reflects a simultaneous increase in both material stiffness and strength, as well as a gradual enhancement of brittle behavior. Under the same curing age conditions, a systematic evolution in the morphology of the stress–strain curves was also induced by an increase in strain rate. As the strain rate increased, the ascending and descending segments of the curves became steeper, which indicated that the stress and elastic modulus rose accordingly, while the strain values decreased correspondingly.

#### 3.1.5. Failure Mode

**Figure 10:**
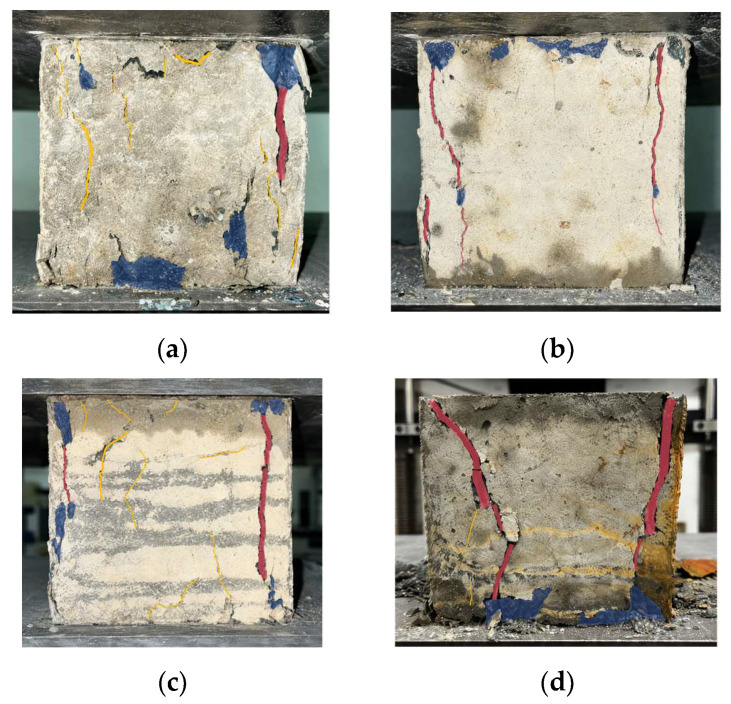
Specimens failure pattern (*T* = 150 d). (**a**) $\dot{\varepsilon }$ = 10^−5^/s. (**b**) $\dot{\varepsilon }$ = 10^−4^/s. (**c**) $\dot{\varepsilon }$ = 10^−3^/s. (**d**) $\dot{\varepsilon }$ = 10^−2^/s.

Figure 10 illustrates the compression failure modes of a BFS35 specimen that had been cured for 150 d and tested at various strain rates. At strain rates of 10^−5^/s and 10^−4^/s, the relatively slow loading rate prolonged the failure process. Numerous dense, short longitudinal microcracks appeared on the specimen surface, and in some areas, mortar spalled off, allowing the microcracks to propagate fully through the interfacial transition zone. As shown in Figure 10, Figure 10a depicts the failure pattern of a specimen tested at $\dot{\varepsilon }$ = 10^−5^/s. On the right side, there is a through-crack (2.2–3 mm wide) that accounts for nearly half of the total crack area; on the left and in the center, there are multiple vertical and oblique microcracks (0.2–0.5 mm wide) that are interconnected in a dendritic pattern; there are approximately 15 effective cracks, with the longest extending to about 90% of the height. Figure 10b shows the failure pattern of a specimen at $\dot{\varepsilon }$ = 10^−4^/s. Cracks are concentrated on both sides, forming two approximately symmetrical vertical cracks with widths of approximately 1.3–1.8 mm; secondary microcracks are approximately 0.2–0.6 mm wide; and localized crushing and spalling occur at the top. At strain rates of $\dot{\varepsilon }$ = 10^−3^/s and $\dot{\varepsilon }$ = 10^−2^/s, the number of surface microcracks on the specimens decreased significantly after damage, with the appearance of penetrating cracks and aggregate spalling. Higher strain rates suppressed the surface propagation of microcracks, and part of the load was borne by the aggregate, resulting in fewer surface cracks. Figure 10c shows the failure pattern of a specimen tested at $\dot{\varepsilon }$ = 10^−3^/s. On the right side, there is a single through-crack extending from top to bottom, 1.8–2.5 mm wide, with approximately 5 effective cracks; the longest of these accounts for about 95% of the height. Figure 10d shows the failure pattern of a specimen at $\dot{\varepsilon }$ = 10^−2^/s, with a diagonal through-crack on the left side (approximately 1.5–6 mm wide, extending from the upper left to the bottom), and a vertical through-crack on the right (approximately 1–5.5 mm wide, with severe spalling at the edges and internal fragmentation), forming two main damage pathways that penetrate the cross-section. There are approximately 4 effective cracks, with the longest extending to about 98% of the height.

### 3.2. NMR Results Analysis

**Figure 11:**
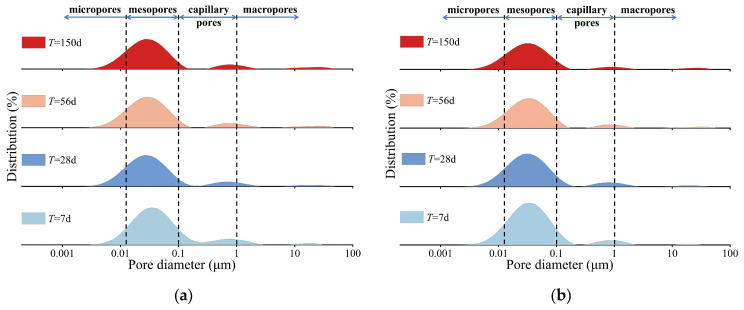
Pore size distribution of RAC. (**a**) *B* = 0%. (**b**) *B* = 35%.

**Figure 12:**
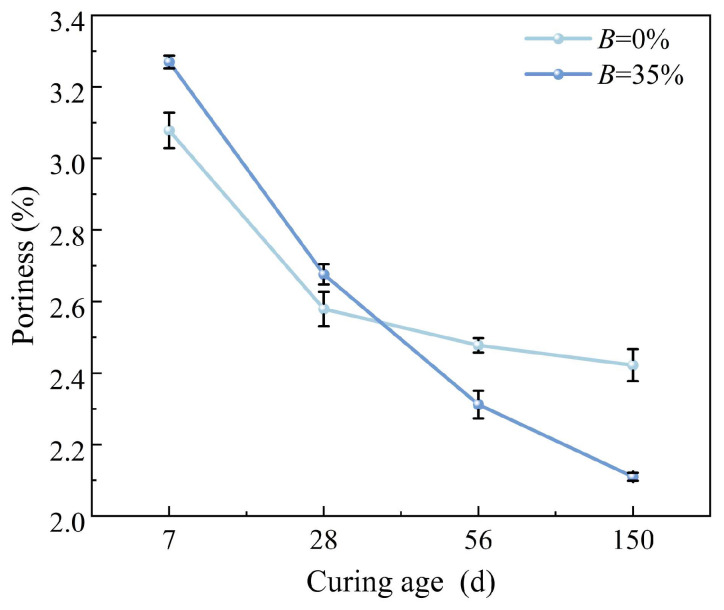
Porosity of RAC.

Figure 11 presents the pore size distribution of RAC. The pore sizes of all specimens were predominantly between 0.01 and 0.1 μm, with the majority being small-sized pores. Figure 12 displays the porosity of RAC. The impact of curing ages on the smaller pores within the specimens was noted to be more significant, displaying a marked reduction as the curing age advanced. This observation indicated that, with the progression of internal hydration, the porosity of the specimens diminished significantly, while the internal compactness exhibited an increase. The reduction in porosity was predominantly attributed to a decrease in small pores [47,48,49]. The findings indicated that the reactivity of GGBFS was progressively activated as the curing age extended. In a subsequent phase, an increased production of C-S-H gels occurred, resulting in a progressive decrease in both pore volume and the number of microcracks. Consequently, it was elucidated that the strength of BFS35 specimens was enhanced in all subsequent stages, demonstrating favorable mechanical properties [50].

**Figure 13:**
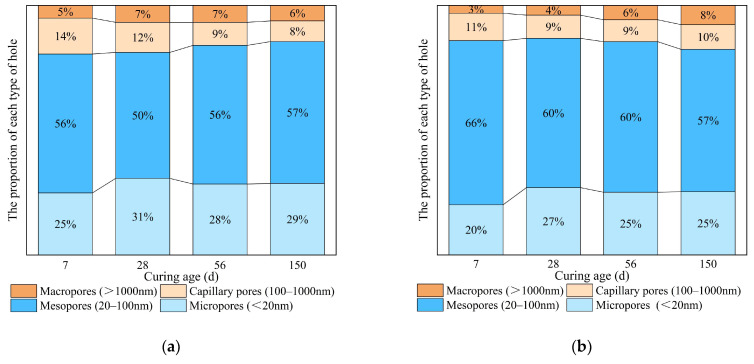
Distribution of hole diameters in RAC specimens. (**a**) *B* = 0%. (**b**) *B* = 35%.

Figure 13 presents the pore size percentages of various RAC specimens [51]. Capillary pores and macropores are harmful pores, and micropores and mesopores are harmless pores. Overall, as the curing age increased, the proportion of harmful pores in the BFS0 specimens decreased, while the proportion of harmless pores increased; in contrast, the proportion of harmful pores in the BFS35 specimens showed an upward trend, while the proportion of harmless pores showed a downward trend. It is worth noting that the C-S-H gel formed by the secondary hydration of GGBFS in the BFS35 specimens acts to fill the pores, resulting in a decrease in the absolute volume of both harmful and harmless pores. However, since the volume of harmless pores decreased to a greater extent than that of harmful pores, this ultimately led to an increase in the proportion of harmful pores.

### 3.3. SEM Results Analysis

**Figure 14:**
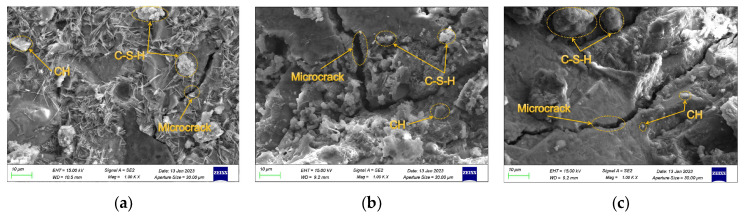
SEM image of BFS0 specimens. (**a**) *T* = 7 d. (**b**) *T* = 28 d. (**c**) *T* = 150 d.

**Figure 15:**
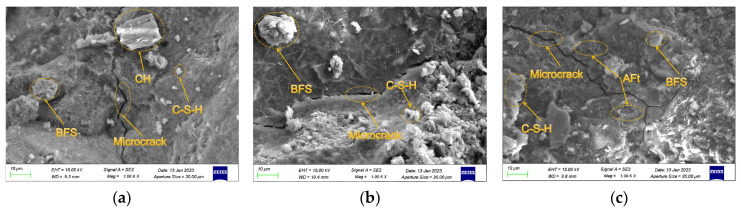
SEM image of BFS35 specimens (**a**) *T* = 7 d. (**b**) *T* = 28 d. (**c**) *T* = 150 d.

Figure 14 and Figure 15 present the SEM results of various RAC specimens. The analysis of these specimens revealed a high concentration of C-S-H gels and CH, accompanied by the presence of significant microcracks. As the curing age advanced, there was an increase in the number and density of hydration products [52]. From Figure 15c, a large amount of C-S-H gels existed and there was no obvious crack, which made the structure denser. The findings suggested that the addition of GGBFS induced a secondary hydration process. The CH underwent a reaction with GGBFS, resulting in the formation of more C-S-H gels, which in turn strengthened the internal structures. Simultaneously, it can be seen that needle and rod-like products appeared, which were inferred to be ettringite (AFt). It has been demonstrated that the incorporation of GGBFS has been shown to promote hydration reactions and generate a greater number of hydration products [52,53,54].

## 4. Analysis of Mesoscopic Damage Mechanism

### 4.1. Statistical Damage Model

Based on the theory of damage evolution-induced catastrophic failure, the failure of quasi-brittle solids such as concrete is divided into two stages by Bai et al. [55]: distributed damage accumulation and induced local catastrophic failure, in which the transition point (critical state) plays a crucial role. It has been confirmed by correlation test results involving deformation localization [56], acoustic emission [57] and electrical resistivity [58] that the critical state of concrete exhibits significant sensitivity characteristics. It is also found that this critical point lags behind the peak nominal stress and is located in the softening region of the nominal stress–strain curve, a phenomenon that differed from conventional understanding.

**Figure 16:**
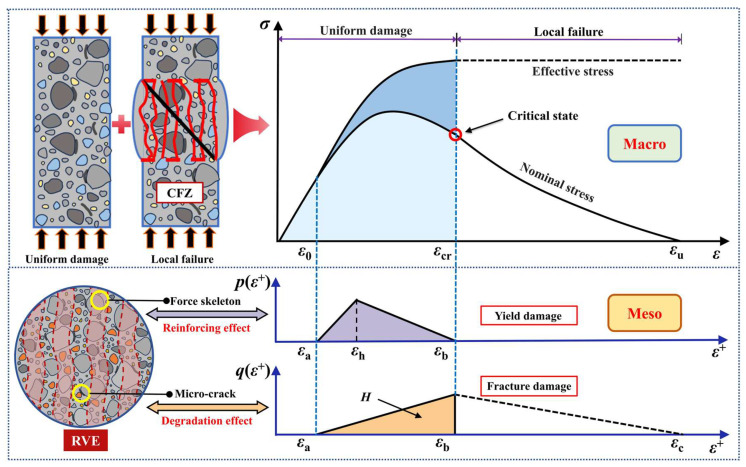
Meso-statistical damage constitutive model for uniaxial compression.

In this study, the uniaxial meso-statistical damage constitutive model of concrete developed by Bai et al. [9,27,34] was used, as shown in Figure 16. The whole uniaxial compression process is divided into two stages: uniform damage and local failure, where the transition point is identified as the critical state. The nominal stress–strain curves and predicted effective stress–strain curves under uniaxial compression are given. The macroscopic nonlinear stress–strain behavior is controlled by two damage modes (fracture and yield) at the fine scale, which represent microcrack initiation and extension, along with the optimization and adjustment of the stress framework in the microstructure, respectively. σ, $\sigma_{E}$ and ε are the nominal stress, effective stress, and strain in the compression direction. $\varepsilon^{+}$ represents equivalent transfer tensile strain ($\varepsilon^{+}\;=-v\varepsilon$, v is Poisson’s ratio, taken as 0.2). In the real situation, *q*(*ε*^+^) and *p*(*ε*^+^) may obey complex statistical distribution laws, such as Weibull, normal and other distribution forms. Considering the complexity of the problem, it can be assumed that both *q*(*ε*^+^) and *p*(*ε*^+^) obey the triangular distribution form in the specific analysis. Through a large number of data analyses, it is shown *q*(*ε*^+^) and *p*(*ε*^+^) can be well fitted to the real stress–strain test curves by adopting the simple triangular distribution form, and can reflect the mechanism of the mesoscopic inhomogeneous damage evolution.

In uniform damage stage, the principal constitutive relations are as follows:

(2) $$\sigma \;=\;E(1-D_{y})(1-D_{R})\varepsilon$$

(3) $$\sigma_{E}=E(1-D_{y})\varepsilon$$

(4) $$D_{y}=\int_{0}^{\varepsilon^{+}}p\left(\varepsilon^{+}\right)d\varepsilon^{+}-\frac{\int_{0}^{\varepsilon^{+}}p(\varepsilon^{+})\varepsilon^{+}d\varepsilon^{+}}{\varepsilon^{+}}$$

(5) $$D_{R}=\int_{0}^{\varepsilon^{+}}q(\varepsilon^{+})d\varepsilon^{+}$$

(6) $$E_{v}=\int_{0}^{\varepsilon^{+}}p(\varepsilon^{+})d\varepsilon^{+}$$

(7) $$p\left(\varepsilon^{+}\right)=\left\{\begin{array}{cc} 0 & \left(\varepsilon^{+}\leq \varepsilon_{a}\right)\\ \frac{2\left(\varepsilon^{+}-\varepsilon_{a}\right)}{\left(\varepsilon_{h}-\varepsilon_{a}\right)\left(\varepsilon_{b}-\varepsilon_{a}\right)} & \left(\varepsilon_{a}<\varepsilon^{+}\leq \varepsilon_{h}\right)\\ \frac{2\left(\varepsilon_{b}-\varepsilon^{+}\right)}{\left(\varepsilon_{b}-\varepsilon_{h}\right)\left(\varepsilon_{b}-\varepsilon_{a}\right)} & \left(\varepsilon_{h}<\varepsilon^{+}\leq \varepsilon_{b}\right) \end{array}\right.$$

(8) $$q\left(\varepsilon^{+}\right)=\left\{\begin{array}{cc} 0 & \left(\varepsilon^{+}\leq \varepsilon_{a}\right)\\ \frac{2H\left(\varepsilon^{+}-\varepsilon_{a}\right)}{{\left(\varepsilon_{b}-\varepsilon_{a}\right)}^{2}} & \left(\varepsilon_{a}<\varepsilon^{+}\leq \varepsilon_{b}\right) \end{array}\right.$$

(9) $$H=D_{R}(\varepsilon_{b})$$

where E represents initial modulus of elasticity. $D_{y}$ represents yield damage. $D_{R}$ represents fracture damage. $E_{v}$ represents evolutionary factor. $\varepsilon_{\mathrm{cr}}$ represents critical state corresponding to compressive strain. *ε*_a_ is the initial damage strain, corresponding to the elastic limit state on the stress–strain curve. *ε*_b_ is the characteristic strain corresponding to the critical state, at which achieves the transition from the distributed damage to the local disaster. *ε*_h_ is the characteristic strain corresponding to the peak point of triangular distribution of $p(\varepsilon^{+})$, which affects the shape of the stress–strain curve. *H* is the fracture damage value corresponding to critical state, equals to the area covered by the function $q(\varepsilon^{+})$ within the interval [*ε*_a_, *ε*_b_].

### 4.2. Analysis of Damage Mechanism

The mesoscopic damage evolution process in RAC is altered by varying strain rates. It should be noted that the mesoscopic damage evolution characteristics of the BFS0 and BFS35 specimens in this study are generally similar; therefore, to maintain the focus and conciseness of the discussion, the mesoscopic damage analysis section focuses primarily on the BFS35 specimen.

As shown in Table 5, damage characteristic parameters which can represent the evolution process were calculated and analyzed using typical stress–strain curves in combination with the preceding paragraph model. For specific steps on determining model parameters, refer to the following references [59]. $R_{E,D}\;=\;\frac{E_{D,i}}{E_{D,0}}$, $R_{E,D}$ represents the influence factor for the elastic modulus under different strain rates. $E_{D,i}$ represents the elastic modulus of the specimen under different strain rates. $E_{D,0}$ represents the elastic modulus of the reference group ($\dot{\varepsilon }$ = 10^−5^/s) under different strain rates.

**Figure 17:**
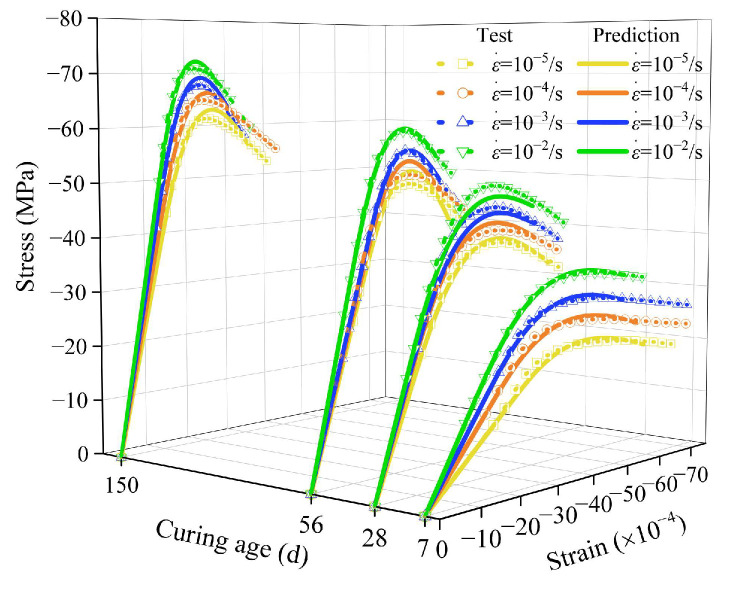
Nominal stress–strain curves of BFS35 specimens.

**Figure 18:**
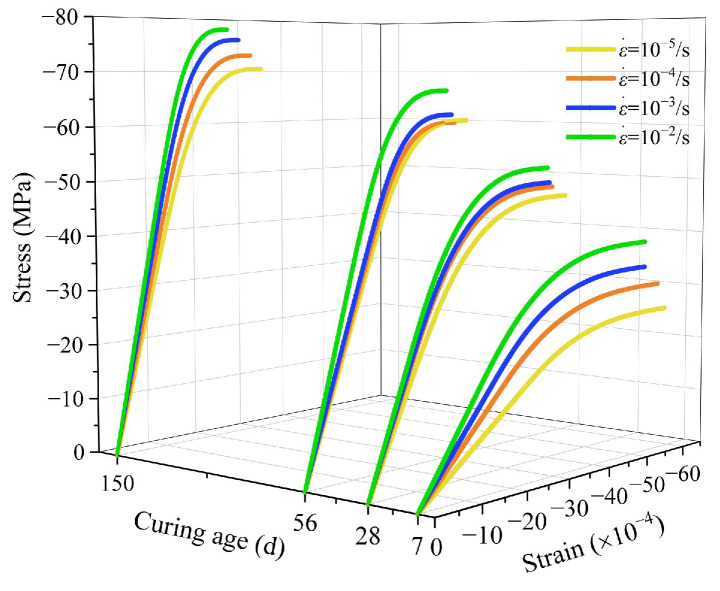
Effective stress–strain curves of BFS35 specimens.

Figure 17 compares the experimentally measured stress–strain curves at various curing ages and strain rates with the corresponding model predictions, demonstrating good agreement between the simulations and the experimental data. Figure 18 presents the effective stress–strain curves obtained from the damage model. During the uniform damage stage, a steady rise in effective stress was observed, and a peak was reached at the critical state point. Upon attaining the critical state, the local failure stage was instantaneously initiated by the BFS35 specimen. As demonstrated in Figure 18, an increase in strain rate resulted in a corresponding rise in stress, indicative of the critical state, accompanied by a decline in strain.

**Figure 19:**
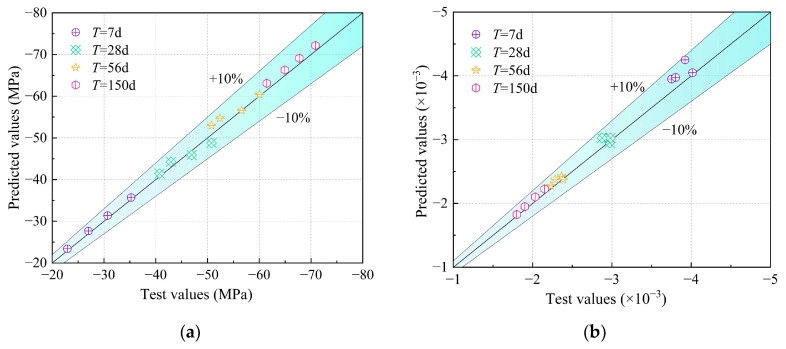
Comparison of experimental results with model predictions. (**a**) Peak stress. (**b**) Peak strain.

Figure 19 shows that the deviations between the predicted and experimentally measured peak stress and peak strain of the BFS35 specimens were below 10%. In general, the model predictions demonstrated a high degree of congruence with the experimental data.

**Figure 20:**
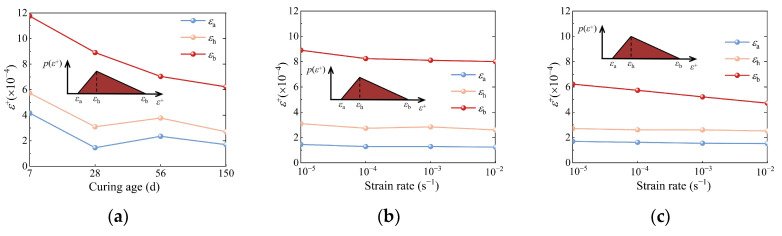
The influence of curing age and strain rate on characteristic parameters $\varepsilon_{a}$, $\varepsilon_{h}$, $\varepsilon_{b}$. (**a**) curing age ($\dot{\varepsilon }$ = 10^−5^/s). (**b**) strain rate (*T* = 28 d). (**c**) strain rate (*T* = 150 d).

The relationship between mesoscopic damage parameters and curing age under quasi-static strain rates is presented in Figure 20a. As the curing age increased, a gradual decrease in the spacing between the mesoscopic damage parameters was observed. It was indicated by this narrowing of the spacing that the transitions between damage stages became more compact, which reflected that the accumulation and evolution of internal damage within the specimen accelerated as the curing age increased.

The relationship between the mesoscopic damage parameters of BFS35 specimens and the strain rate at different curing ages is illustrated in Figure 20b,c. Taking *T* = 28 d as an example, as the strain rate was increased from $\dot{\varepsilon }$ = 10^−5^/s to $\dot{\varepsilon }$ = 10^−2^/s, the characteristic parameter $\varepsilon_{a}$ of the BFS35 specimen was observed to decrease from 1.456 × 10^−4^ to 1.244 × 10^−4^; $\varepsilon_{h}$ decreased from 3.098 × 10^−4^ to 2.602 × 10^−4^; and $\varepsilon_{b}$ decreased from 8.908 × 10^−4^ to 8.006 × 10^−4^. Thus, similar trends were exhibited by these characteristic parameters. As the strain rate increased, a decreasing trend was shown by the parameters of specimens at all curing ages. Furthermore, it was also shown in Figure 20 that, among these three characteristic parameters, the distance between $\varepsilon_{a}$ and $\varepsilon_{h}$ was smaller than that between $\varepsilon_{b}$ and $\varepsilon_{h}$.

**Figure 21:**
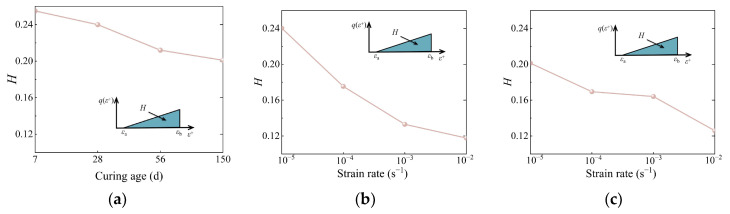
The influence of curing age and strain rate on characteristic parameters *H.* (**a**) curing age ($\dot{\varepsilon }$ = 10^−5^/s). (**b**) strain rate (*T* = 28 d). (**c**) strain rate (*T* = 150 d).

The evolution curve of the key parameter *H* (representing fracture damage) in BFS35 specimens under quasi-static strain rate conditions is presented as a function of curing age in Figure 21a. A decreasing trend in the *H* value with curing age was observed for the specimens. Since the extent of fracture damage is related to microcrack density, it was indicated by the pattern of change in the *H* value that, during uniaxial compression, the microcrack density at which the BFS35 specimens reached a critical state tended to decrease as the curing age increased.

The relationship between the parameter *H* and the strain rate for BFS35 specimens at different curing ages is presented in Figure 21b,c. Taking the BFS35 specimen at *T* = 28 d as an example, when the strain rate increased from $\dot{\varepsilon }$ = 10^−5^/s to $\dot{\varepsilon }$ = 10^−2^/s, the fracture damage value *H* at the critical state decreased from 0.240 to 0.118. Since *H* is directly related to microcrack density, this indicates that as the strain rate increases, the microcrack density required for the specimen to reach the critical state gradually decreases. The magnitude of the *H* value can be qualitatively evaluated based on the appearance of the cracks on the specimen after failure, and its trend is consistent with the observed density of surface failure cracks. In a similar manner, an increasing strain rate was observed to be associated with a decreasing trend in the parameters of the specimens at all curing ages. The observed trend indicated that as the strain rate increased, the density of microcracks within the specimens decreased, which was consistent with the observed test behavior.

**Figure 22:**
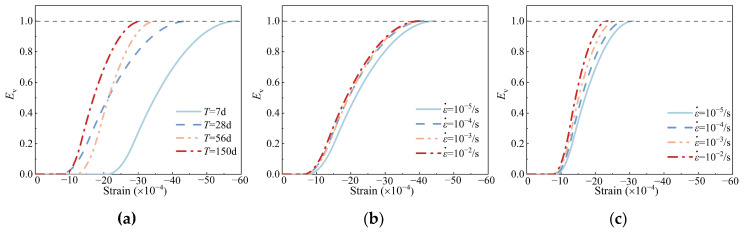
The influence of curing age and strain rate on $E_{v}\;$ evolution curves. (**a**) curing age ($\dot{\varepsilon }$ = 10^−5^/s). (**b**) strain rate (*T* = 28 d). (**c**) strain rate (*T* = 150 d).

Figure 22a illustrates the evolution of the factor $E_{v}$ with curing age for BFS35 specimens under quasi-static strain rate conditions. $E_{v}$ quantifies the extent to which the microstructural stress framework undergoes optimization and adjustment. During the uniform damage stage, $E_{v}$ is observed to vary between 0 and 1, and it plays a critical role in the overall damage evolution process. The results indicated that under quasi-static loading conditions, with increasing curing age, significant differences in the $E_{v}$ evolution process among the specimens became apparent, which reflected that the optimization and adjustment process of the microstructural stress framework was significantly impacted by curing age.

Figure 22b,c present the variation of the evolution factor ($E_{v}$) as a function of strain rate for the BFS35 specimens. Taking *T* = 28 d as an example, as the strain rate was increased, the $E_{v}$ evolution curve of the BFS35 specimen was observed to become steeper, and a gradual decreasing trend was shown by the strain corresponding to the initial damage. As the strain rate increased, the $E_{v}$ evolution curves of all the specimens at different curing ages showed the same trend. These results indicated that the evolution of $E_{v}$ in the specimens was significantly accelerated by higher strain rates (compared to quasi-static). From the perspective of deformation, as the strain rate increased, a corresponding decrease in the critical strain, a reduction in ductility, and a weakening of the ability to resist deformation were observed in the specimens.

**Figure 23:**
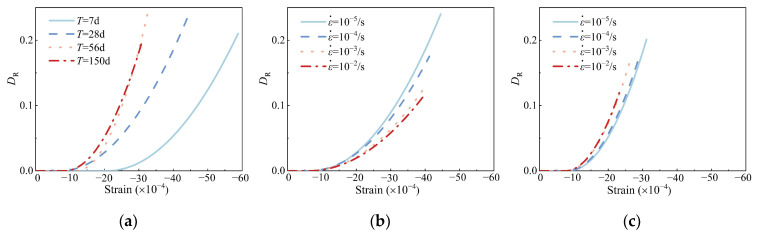
The influence of curing age and strain rate on $D_{R}$ evolution curves. (**a**) curing age ($\dot{\varepsilon }$ = 10^−5^/s). (**b**) strain rate (*T* = 28 d). (**c**) strain rate (*T* = 150 d).

Figure 23a depicts the variation of the fracture damage parameter $D_{R}$ with curing age for BFS35 specimens under quasi-static loading. The $D_{R}$ coefficient quantifies the formation and growth of microcracks inside the specimen. The results indicated that under quasi-static loading conditions, as the curing age increased, the $D_{R}$ evolution process for each specimen was completed within a narrower strain range. It was observed that damage accumulated more rapidly in specimens that had undergone a longer curing period. Furthermore, it was determined that the critical damage state was attained even in cases of relatively minor macroscopic deformations.

Figure 23b,c show the evolution of the fracture damage variable $D_{R}$ for the BFS35 specimens subjected to various strain rates. Taking *T* = 28 d as an example, as the strain rate was increased, the transition from initial damage to local failure was completed by the $D_{R}$ evolution curve of the BFS35 specimens within a narrower strain range. As the strain rate increased, the $D_{R}$ evolution curves of all the specimens at different curing ages showed the same trend. This phenomenon was attributed to the strain rate sensitivity of the formation and propagation rates of microcracks within the specimens.

**Figure 24:**
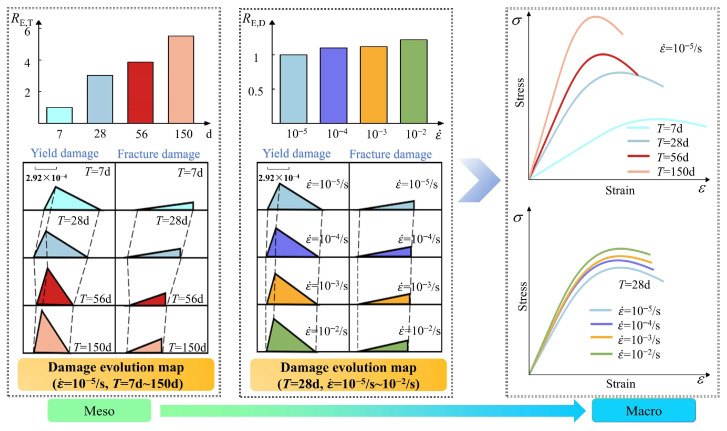
Relationship between macroscopic stress–strain behavior and mesoscopic damage mechanisms.

The macroscopic nonlinear stress–strain behavior of concrete, as illustrated in Figure 24, depended on the initial mechanical properties of the material and its damage evolution process during compression. In this context, $R_{E,T}\;=\;\frac{E_{T,i}}{E_{T,0}}$, where $R_{E,T}$ represents the influence factor for the elastic modulus under curing age. $E_{T,i}$ represents the elastic modulus of the specimen under different curing ages. $E_{T,0}$ represents the elastic modulus of the reference group (*T* = 7 d) under curing age. The elastic modulus reflected the initial mechanical properties of the material, while the damage evolution process reflected the fracture and yield damage mechanisms at the microscopic level. In terms of deformation characteristics, the overall shape of the triangular probability density distributions of $p(\varepsilon^{+})$ and $q(\varepsilon^{+})$ was shifted to the left. These micro-level changes were ultimately manifested at the macro-level as a gradual increase in peak stress, a gradual decrease in peak strain, and a steepening of the descending segment of the stress–strain curve with increasing curing age or strain rate.

## 5. Conclusions

This study employed a combined methodology of uniaxial compression tests, microstructural analysis, and theoretical analysis to investigate the effects of curing age and strain rate on the mechanical properties and mesoscopic damage mechanisms of GGBFS-modified RAC. The specific findings are as follows:

1. Under quasi-static loading conditions ($\dot{\varepsilon }$ = 10^−5^/s), the peak stress of the BFS35 specimen remained lower than that of the BFS0 specimen until *T* = 56 d. For example, at *T* = 28 d, the peak stress of the BFS35 specimen was −40.69 MPa, while that of the BFS0 specimen was −41.75 MPa-still 2.54% lower. However, once the curing age exceeded 56 days, the BFS35 specimen surpassed the BFS0 specimen. For example, at *T* = 56 d, the peak stress of the BFS35 specimen was −50.37 MPa, while that of the BFS0 specimen was −48.37 MPa, an increase of 4.13%. This indicates that GGBFS was activated during the later stages of curing, effectively enhancing the macroscopic mechanical properties of the specimens.
2. As the curing age increased, the rate of increase in the dynamic strength enhancement factor (DIF) showed a decreasing trend. The increase in DIF for the BFS0 specimens (from $\dot{\varepsilon }$ = 10^−5^/s to $\dot{\varepsilon }$ = 10^−2^/s) decreased from 11.4% at *T* = 7 d to 6.14% at *T* = 150 d. In contrast, the increase in DIF for the BFS35 specimens reached as high as 19.1% in the early stage (*T* = 7 d), which was significantly higher than that of the BFS0 specimens during the same period. As the curing age increased to 150 days, the DIF increase for the BFS35 specimen decreased to 4.97%, which was lower than the 6.14% observed for the BFS0 specimen. This indicates that the addition of GGBFS significantly enhanced the strain rate sensitivity of RAC in the early stages, but this sensitivity deteriorated as the curing age increased.
3. When the curing age was 150 days, it was observed that the loading rate was lower for the BFS35 specimens at a low strain rate. The specimens required a greater time to failure, and the microcracks were fully extended from the weak ITZ, followed by the formation of additional microcracks. Conversely, at the higher strain rates (compared to quasi-static), the loading speed was faster. The microcracks were unable to be extended in time, and the aggregate helped to bear part of the load, thereby reducing the surface microcracks.
4. By carrying out NMR and SEM tests, it was observed that the hydration reaction was more adequate and the internal organization of the BFS35 specimens became more and more compact as the curing age was extended. During the later stages of curing, the active components of GGBFS were activated, reacting with Ca(OH)_2_ to form additional C-S-H gel, which filled the pores, increased density, and reduced porosity, thereby improving the macroscopic initial mechanical properties of the BFS35 specimens, which are primarily reflected in the elastic modulus.
5. The two triangular probability density distributions in the damage evolution diagram are governed by four characteristic parameters (*ε*_a_, *ε*_h_, *ε*_b_, *H*), and their morphological changes directly reflect the complete evolution process from microcrack initiation and propagation to through-cracking. Taking the BFS35 specimen at *T* = 28 d as an example, when the strain rate increased from $\dot{\varepsilon }$ = 10^−5^/s to $\dot{\varepsilon }$ = 10^−2^/s, the *ε*_a_, *ε*_h_ and *ε*_b_ decreased from 1.456 × 10^−4^, 3.098 × 10^−4^ and 8.908 × 10^−4^ to 1.244 × 10^−4^, 2.602 × 10^−4^ and 8.006 × 10^−4^, respectively. At the same time, the fracture damage value *H* at the critical state decreased from 0.240 to 0.118. This indicates that as the strain rate increased, the microcrack density required for the specimen to reach the critical state gradually decreased.
6. This study reveals the evolution of mesoscopic damage in GGBFS-modified RAC under different curing ages and strain rates, providing a theoretical basis for its engineering applications. Due to experimental limitations, this study did not conduct quantitative analyses such as EDS, nor did it determine the sand content in the bonding mortar of the RAC; systematic research on these aspects will be conducted in the future.

## Figures and Tables

**Table 1:** Main components of cement and GGBFS.

Material	CaO (%)	SiO_2_(%)	Al_2_O_3_(%)	MgO (%)
Cement	53.49	23.88	9.54	4.49
GGBFS	35.58	36.1	16.32	11.32

**Table 2:** Performance index of RCA.

Aggregate Size (mm)	Apparent Density (kg/m^3^)	Water Absorption (%)	Moisture Content (%)	Void Ratio (%)	Crushing Index (%)
5–20	2590	4.2	3.5	41	13.6

**Table 3:** Design of RAC (kg/m^3^).

Type	Water-Binder Ratio	Cement	Sand	GGBFS	RCA	Water	Water Reducer	Additional Water
BFS0	0.46	360	646	0	1228	166	0.9	8.6
BFS35	0.46	234	646	126	1228	166	0.9	8.6

**Table 4:** Summary of the number of test specimens.

Number	Number of Groups	Quantity per Set	Curing Age	Strain Rate	Total
BFS0	6	4	7 d, 14 d, 28 d, 56 d, 90 d, 150 d	3 × 10^−5^/s	24
BFS35	6	4	7 d, 14 d, 28 d, 56 d, 90 d, 150 d	3 × 10^−5^/s	24
BFS0	16	4	7 d, 28 d, 56 d, 150 d	10^−5^/s, 10^−4^/s, 10^−3^/s, 10^−2^/s	64
BFS35	16	4	7 d, 28 d, 56 d, 150 d	10^−5^/s, 10^−4^/s, 10^−3^/s, 10^−2^/s	64

**Table 5:** Damage characteristic parameters.

Group (BFS35)	$\dot{\varepsilon }$/s	*R*_E,D_	$\varepsilon_{a}$/×10^−4^	$\varepsilon_{h}$/×10^−4^	$\varepsilon_{b}$/×10^−4^	*H*	R^2^
*T* = 7 d	10^−5^	1	4.174	5.734	11.758	0.255	0.9869
	10^−4^	1.35	3.088	4.537	11.352	0.196	0.9921
	10^−3^	1.61	2.651	4.422	10.627	0.148	0.9959
	10^−2^	1.95	1.992	3.960	10.620	0.132	0.9992
*T* = 28 d	10^−5^	1	1.456	3.098	8.908	0.240	0.9963
	10^−4^	1.10	1.288	2.735	8.247	0.175	0.9963
	10^−3^	1.12	1.288	2.839	8.108	0.133	0.9960
	10^−2^	1.22	1.244	2.602	8.006	0.118	0.9982
*T* = 56 d	10^−5^	1	2.352	3.783	7.037	0.212	0.9869
	10^−4^	1.05	2.397	3.586	6.486	0.207	0.9911
	10^−3^	1.09	2.395	3.588	6.347	0.179	0.9993
	10^−2^	1.34	1.607	3.022	6.102	0.163	0.9991
*T* = 150 d	10^−5^	1	1.697	2.707	6.223	0.201	0.9907
	10^−4^	1.11	1.622	2.611	5.736	0.169	0.9956
	10^−3^	1.22	1.548	2.603	5.215	0.164	0.9979
	10^−2^	1.34	1.523	2.523	4.719	0.126	0.9972

## Data Availability

The original contributions presented in this study are included in the article. Further inquiries can be directed to the corresponding authors.
